# Challenging the status quo: gender norms in Croatian juvenile correctional settings

**DOI:** 10.3389/fsoc.2024.1411894

**Published:** 2024-06-26

**Authors:** Elizabeta Matković, Ivana Borić, Andrea Ćosić, Anamarija Sočo

**Affiliations:** ^1^Status M, Zagreb, Croatia; ^2^Department of Behavioural Disorders, Faculty of Education and Rehabilitation Sciences, University of Zagreb, Zagreb, Croatia

**Keywords:** gender norms, juveniles, correctional institutions, treatment programs, civil sector

## Abstract

Gender norms and issues related to gender are highly relevant when it comes to treatment of juveniles in correctional institutions, especially related to their risky behavior and personal characteristics (such as personality traits, intellectual capability, culture, ethnicity etc.). Furthermore, many juveniles in correctional institutions are exposed to violence and are also perpetrators of violent criminal acts. This paper will give an overview of national policies related to gender-sensitive treatment in Croatia as a background to research aimed to describe the reflection of gender issues and gender norms in practical work with juveniles in Croatian correctional institutions. The research is based on qualitative approach and includes focus groups with youth workers from various organizations who implemented programs for youth in correctional institutions. Preliminary results show that gender and gender norms are not specifically targeted in treatment programs within the institutions. The initiative to implement gender issues mainly comes from organizations from the civil sector through different workshops. Juveniles express relatively rigid gender norms that are supported by rigid organization of correctional institutions especially for males. This research shows the importance of clearer focus to gender-sensitive programing and gender sensitive treatment programs that will strongly be integrated in everyday practice of correctional institutions. The precondition for this is largely connected to deconstructing stereotypes about gender and gender norms both for youth and professionals working with them.

## Introduction

The System of Justice is a complex entity aimed at ensuring the conditions necessary for the quality functioning of the legal order in the Republic of Croatia, as well as preserving its fundamental values. Within this system, children and young people may find themselves either in the position of a victim or that of a perpetrator, though it is not uncommon for them to occupy both roles simultaneously. For the purposes of this paper, we will focus on children and young people who committed a criminal offense or misdemeanor and can be held criminally responsible.

Juvenile Courts Act (2019; hereinafter: the JCA) in Croatia stipulates that individuals up to 14 years of age are considered children and cannot be held criminally responsible. Those aged between 14 and 18 are classified as minors (with 14–16 being younger minors and 16–18 older minors) and are criminally liable. The JCA also extends its applicability to individuals aged 18 to 21, referred to as younger adults. It outlines the treatments available, which are educational and correctional measures, including court reprimand; special obligations and duties; intense care and supervision; intense care and supervision with day stay at an educational institution; referral to a disciplinary center; referral to an educational institution; referral to a correctional institution; and referral to a special educational institution. Specifically, the measure of referral to a correctional institution, as defined by the JCA, mandates court orders when necessary to separate the minor from their environment to apply intense educational measures due to significant behavioral disorders and an insufficient willingness to accept educational influences. In deciding on this measure, the court considers the gravity and nature of the offence committed and whether educational measures or a juvenile prison sentence were previously imposed on the minor.

In Croatia, correctional institutions fall under the jurisdiction of the Ministry of Justice and Public Administration, akin to adult penitentiaries. These institutions for girls and boys are, in fact, situated near adult prisons. Out of a total of 274 convicted minors in 2022, 74 minors were referred to a juvenile institution (Ministry of Justice and Public Administration, 2024). Out of the 74 minors, 57 male minors and 17 female minors were detained in a juvenile institution, which is roughly the number of minors undergoing this measure in 2021 (59 male minors and 16 female minors). In 2022, among the young men and women in juvenile institutions, property crimes predominated at 66.67%, followed by crimes against personal freedom at 17.78%, crimes against life and body at 8.89%, and crimes against marriage, family, and children, crimes of sexual abuse and exploitation of children, and crimes against sexual freedom, each represented at 6.66% (Ministry of Justice and Public Administration, 2024). The relatively low figures are in line with the general trends in the dynamics of juvenile crime in Croatia, which has shown a downward trend in the last 20 years (Borić and Ćosić, 2022; Ricijaš et al., 2022).

A recent survey paints a picture of typical minors in a correctional institution in Croatia: 16-year-old boys detained because of a theft or burglary and described by their caseworkers as having the lower intellectual ability, disturbed family relations, a history of criminal offenses and mental health problems. Similarly, the “typical girl” is described as having low self-confidence and poor self-image, aggressiveness, underdeveloped empathy and below-average intellectual ability. Most of the young people grow up in unsupportive families which are often engaged with or under the supervision of social work agencies. Additionally, minors often come to the correctional institution from other institutional settings within the child protection system (Borić and Ćosić, 2022).

Young Roma people, particularly men, are significantly overrepresented in juvenile institutions in Croatia. This echoes the troubling patterns seen in the overrepresentation of Black, Roma and Gypsy children in child protection services globally (Cénat et al., 2021; Allen and Hamnett, 2022). This overrepresentation can be attributed to a confluence of factors that, while complex, often find common ground in the issues of unfavorable socio-economic factors present in the minority population, and institutional discrimination based on ethnicity/race which is deeply embedded within the decision-making processes of juvenile justice systems (Hill, 2004). The analysis and response to such disparities necessitate an intersectional approach, considering the intertwined dimensions of gender, ethnicity/race and socioeconomic status. Acknowledging and addressing these overlapping systems of discrimination is crucial in devising more equitable and just juvenile justice policies and practices.

Historically, the juvenile justice system has employed a one-size-fits-all care model that fails to recognize different service needs between genders (Chesney-Lind and Shelden, 2013; Galardi and Settersten, 2018). Despite the acknowledged importance of tailoring treatment to juvenile offenders’ needs, Croatia lacks both gender-sensitive treatment and gender-specific approaches to juvenile justice.

The gender-sensitive approach entails the ability to perceive, feel, differentiate, and acknowledge the existing gender and other differences among children and youths, the presence of discrimination in society (primarily based on gender), displays of inequality, and to incorporate these aspects into pedagogical actions and strategies (Rasskazova et al., 2021). Researchers and practitioners advocate for gender-specific programming for delinquent youth, aimed at addressing the unique risk factors, pathways, and outcomes of delinquency for both young men and young women (Galardi and Settersten, 2018). For policies and interventions to be effective for at-risk youth in detention, they must be gender-sensitive, taking into account the distinct needs and experiences of both young men and young women.

Internalized gender norms and gendered attitudes encompass the process by which individuals adopt and enact socially prescribed gender expectations, especially regarding the roles and behaviors linked to being a man or a woman (Connell and Messerschmidt, 2005). Likewise, gender plays a crucial role in grasping the experiences of detained youth. Studies reveal that detained girls possess distinct needs and experiences, which are frequently overlooked in the design and implementation of policies and interventions (Mears et al., 2004; Moura et al., 2023). Likewise, detained young men may face unique gender-related challenges, such as the expectation to adhere to prevailing masculine norms, potentially leading to aggressive or violent behavior (Flood and Pease, 2009).

Research and theory concerning juvenile (re)offending have neglected to examine how young men’s gender identities are constructed in the context of a juvenile justice system in which behavior reform through various therapies is a primary goal (Abrams et al., 2008). The population in correctional institutions predominantly comprises young men, about 80%, but the gender dimension is overlooked. Both boys and young men, as well as girls, develop a range of gender-based responses to their social environments as they grow (Carr et al., 2008). The role of correctional institutions in shaping young men’s masculinities, closely linked to criminal attitudes and behaviors, remains underexplored (Abrams et al., 2008). The disproportionate referral of young men to correctional institutions raises questions about the gender norms and masculinities to which these young men are exposed. Culturally defined masculine norms influence men’s expressions of anger and aggression (Jakupcak et al., 2005). For some young men, fitting into the defined masculine box means displaying strength, concealing vulnerabilities, and manifesting strength through violence.

Public policies and interventions aimed at detained youth or those working in juvenile institutions in Croatia show an almost total absence or extremely limited application of a gender perspective. Although the significance of gender considerations in public policy is increasingly acknowledged in broader discussions, the practice of incorporating gender perspectives comprehensively across child protection and juvenile justice sectors in Croatia is still lacking (Borić and Ćosić, 2022). Treatment in juvenile institutions in Croatia has yet to fully embrace the critical need for deconstructing rigid gender norms that significantly impact the rehabilitation and development of detained youth. Understanding the ways in which internalized gender norms and masculinities shape individuals’ experiences can help to identify opportunities for intervention and treatment (Levant and Richmond, 2007; Moura et al., 2023).

The professionals working in juvenile institutions are generally not familiar with gender-related issues. They state that they did not have the opportunity to get training on topics related to gender, gender-based violence and gender equality. There is a manifest need to raise their awareness and build competencies around these topics (Borić and Ćosić, 2022).

The current approach to gender issues within these institutions is narrowly confined to occasional workshops. These sessions, facilitated by a select few external associations, represent sporadic attempts to introduce gender-transformative interventions. Such efforts, while valuable, fall short of a comprehensive and integrated strategy required to address the deeply ingrained gender norms that influence the behaviors and identities of young individuals in detention (Borić and Ćosić, 2022). This article will explore how those implementing these programs (educators, workshop facilitators) perceive the prevailing gender norms in correctional institutions and their efforts to address the topic of gender.

### Study aims

The aim of the study was to explore gender norms in Croatian correctional institutions for youth from the perspective of youth workers, external associates of correctional institutions. Three research questions were posed:

1. What are the characteristics of correctional institutions in the context of gender norms?
2. What are the specific characteristics of young people in correctional institutions?
3. How are gender norms reflected in the work with young people in correctional institutions?

## Methods

The data was collected through focus group discussion, allowing participants to share their experiences while reflecting on their own views in relation to others, resulting in a specific synergy of perspectives.

### Participants

The focus group participants were representatives of civil sector organizations with many years of experience in implementing various group programs in correctional institutions. Six youth workers from four associations took part in the study. Most of the participants are experts in the field of psychosocial work - psychologists, social workers, and social pedagogues as well as an artist. The age ranged from 27 to 46 years. In terms of gender, the focus group comprised 4 women and 2 men. The topics of the programs in which the participants worked with young people in correctional institutions relate to gender and gender roles, preparing young people for independent living and creative workshops.

### Data analysis

The data was processed using thematic analysis (Braun and Clarke, 2006) in the following steps: verbatim transcription of the focus group, initial coding, revision of initial codes, definition of themes, final revision of themes in relation to the research aim and research questions. The analysis of the data was carried out by the first two authors of the paper, and the final revision of the themes involved the other two authors of the paper.

Four key themes were defined: (1) the rigid structure of correctional institutions; (2) the gender stereotypical division of correctional institutions; (3) the characteristics of juveniles in correctional institutions; (4) the characteristics of the program of external organizations.

## Results

The aim of the study was to explore gender norms in Croatian correctional institutions for youth from the perspective of youth workers, external associates of correctional institutions. Four key themes were derived from the data: (1) the rigid structure of correctional institutions; (2) the gender stereotypical division of correctional institutions; (3) the characteristics of juveniles in correctional institutions; (4) the characteristics of the program of external organizations.

### The rigid structure of correctional institutions

The first theme refers to the rigidity of the work and life structure in correctional institutions and includes the following codes: high degree of structure and rules, restrictive spatial arrangement of correctional institutions, formality and distance in relations, rigidity of correctional institutions in accepting criticism.

When describing their experiences of working in educational institutions, the focus group participants primarily reflected on the high degree of structure and rules in educational institutions. The high level of structure is already evident when agreeing on the working methods and timetable for the arrival of external youth workers, insisting on adherence to the usual daily routine and the impossibility of changing previously agreed dates. Correctional institutions are part of the justice system and are located in close proximity to prisons for adult offenders. They are closed facilities with very clear rules and procedures. The participants describe that there is a high level of security in the correctional institutions, especially in the male correctional facility. Personal belongings and cell phones must be handed in at the entrance, everything that is to be brought into the correctional institution (e.g., work materials, refreshments for young people) must be registered, and the prior approval of the manager must be obtained for everything. For the youth workers, these procedures were sometimes unpleasant, Participant 1 stated:

“I must admit that this procedure on entering the institution, where you put your cell phone down, for example, is really uncomfortable. It’s uncomfortable for me, and I can only imagine how is it for them…”

In addition, the correctional institution for young men also employs judicial police officers who are required to supervise all aspects of youths’ lives. Some youth workers also state that the encounters with the judicial police officers were sometimes more difficult than those with the juveniles, for example Participant 1 said:

“I was more uncomfortable in front of the judicial police officers than in front of the boys….”

The correctional institutions are restrictively designed spaces. The correctional institution for young men is located inside the adult prison, is surrounded by barbed wire and the windows are barred. Although this correctional institution has been recently renovated and is located in a quiet area with lots of greenery, the restrictive and repressive elements are still visible. The focus group participants warn that the repressive context of the physical facility itself does not meet the needs of young people born and raised in extremely unprivileged circumstances, e.g., Participant1:

“I would remove most of these things of the repressive regime that is imposed on them. Boys and girls who were born in extremely unprivileged circumstances and did not have the opportunity to develop and who were completely wrongly supported. In other words, they are not supported at all, only punished.”

According to the participants, the spatial design of correctional institutions is punishable. The correctional institution for girls is smaller and apparently less restrictive than for young men. However, the space itself is restrictive, as it only has a small capacity and a very small outdoor area. There are no judicial police in the girls’ correctional facility, and security is provided by program staff who are not judicial police officers. In this sense, the experience of oppression is somewhat less.

The physical regulation and restrictiveness of correctional institutions and a high degree of rules and structures are reflected in the formality and distance in relationships, whether in the attitude of staff toward young people or in the attitude of staff toward external youth workers. The participants note that this formality imposed by the correctional institutions also influences the work dynamics and the young people’s relationship with external youth workers. They believe that it is difficult to build closer relationships with the young people as it is insisted that the young people address them formally (“Ma’am, sir”). In addition, in the correctional institution for young men, all activities are supervised by judicial police officers, and in the correctional institution for young women, professional staff supervise activities, and there is a warning that activities “must not talk about staff from the correctional institution.” Some of the participants note that the behavioral and organizational policies of the correctional institutions have a great influence on the work of the external educators, Participant 4 highlighted:

“The policies of the institution, some practices of the institution, generally very much determine how much the young people in the institution will receive something, as much as they trust us.”

In addition, participants note that in some situations they were warned in advance by the staff about young people and their inappropriate behavior before they could meet them themselves, suggesting that young people are in some ways classified as “problematic.”

The distance in relationships is also reflected in relationship of the staff toward external youth workers, and in this sense, participants mention negative experiences and the rigidity of correctional institutions in accepting criticism and objections from external associates. The participants describe situations in which they drew the attention of the management and staff of correctional institutions to the inappropriate treatment of individual staff members toward young people and to situations in which staff members used violence toward young people, whereupon the correctional institutions usually adopted a defensive attitude, reduced the scope of cooperation with external organizations or even terminated the cooperation altogether.

### The gender-stereotypical division of correctional institutions

As described in the first theme, a high degree of structure and rigidity was found in correctional institutions in terms of the physical environment and relationships. In addition, the second theme focuses specifically on the aspect of gender stereotypical arrangements of correctional institutions. Within this theme, the following codes are defined: gender-stereotypical landscape design, gender-stereotypical treatment content provision, gender-stereotypical attitudes toward juveniles, and lack of gender sensitivity among correctional institution staff.

Correctional institutions are structured in a gender-stereotypical way, which was already established in the context of the first theme. The correctional institution for young men has a larger capacity, which is logical considering that in Croatia there are significantly more young men in the justice system than girls (1/3 of girls, 2/3 of boys). The correctional institution for girls has a smaller capacity, there is a smaller number of girls and staff, and the impression is that the environment is less repressive. Participants experience male correctional institution as more repressive due to the presence of the judicial police. The spatial design of the correctional institutions is also linked to the gender-stereotypical treatment facilities, which is particularly evident in the sports facilities. In the correctional institution for young men, there are more sports facilities that are typically geared toward the male population (e.g., football and basketball), and there are larger outdoor sports fields. There is also a gym in the young men’s institution, whereas there is none in the institution for girls.

The facilities in the girls’ correctional institution are designed in such a way that they offer few opportunities for exercise and physical activity. Of the outdoor sports fields, there is only a small volleyball court. The participants report that the girls are occasionally offered activities such as yoga and conscious breathing, which are not available to the young men at the same time. In addition, girls are offered more handicraft content, such as crocheting, which is also perceived as gender-stereotypical by the participants. The general orientation of working with youth in correctional institutions is fundamentally gender-specific and stereotypical, Participant 1 explained:

“Girls work on…. They know how to cook, so they can also crochet. In this sense, they are pressed into this mold, which is traditionally Croatian.”

When choosing the content and methods of the external youth workers, it was suggested to choose “softer” techniques, for example, working with young men with the comic technique and with girls with the technique of anti-stress painting. In this sense, participants believe that the legal system in which correctional institutions operate is stereotyped and that young people are self-directed, i.e., Participant 1:

“…pressed into the birth mold and not allowed to have their own gender expression…”

However, some participants point out that even within the existing gender “molds” some exceptions can be observed, but to a greater extent among young men. For example, they mention several creative activities for young men, such as making mosaics and engaging in other art techniques in established creative studios. There are no such opportunities in the institution for girls. Participants emphasize that such an arrangement and range of treatment programs is outdated and restrictive and point out that the needs of young people cannot be well met in such an environment.

Young people generally question the expected gender roles to a small extent. When they talk about their families, they refer to the traditional gender roles of father and mother. They are not at all familiar with gender issues, gender norms and such content stimulated by programs of external organizations and newspapers. There is no similar content in the correctional institutions. With regard to the girls, the participants note that during the program the girls have learned to realize that not every woman necessarily has to be a mother, especially girls from the Roma national minority. The participants point out that the girls were particularly surprised by the topic of gender-based violence.

With regard to the attitude of correctional institution staff toward young people, external youth workers also note a gender-stereotypical approach and relationship. The approach to girls is much milder and more intimate, it is more emotionally focused and there is more conversation with girls. Girls are encouraged to express feelings, except anger. Expressing anger is often punished, i.e., Participant 1:

“Girls just should not get angry, because if they get angry they are really put in solitary confinement. Because it’s undesirable for girls to be angry.”

Young men are more likely to be seen and labeled as violent by staff in correctional institution, and in staff attitudes toward young men, external youth workers note a tougher approach, but also a greater tolerance of rougher language, swearing and even vulgarity. For girls, such behavior is much less tolerated. For example, drawings made by girls as part of some activities in the programs of external organizations were asked to “cover inappropriate drawings with a collage,” which was not the case in the correctional institution for young men. In terms of staff attitudes toward young people, participants noted that there is a “calm and milder aggression” toward girls, in terms of taking things away or refusing services, whereas the attitude toward young men is harsher. For example, Participant 5 stated:

“I have noticed that they treat boys quite harshly, literally hit them on the back and stuff, they are just quite rough.”

The participants also note that correctional institutions are an “extremely conservative environment” where not much gender diversity or gender expression is allowed. “There are few opportunities to be who you are.”

The participants also noted a lack of gender sensitivity among staff at correctional institutions. In this context, they also referred to the gender of staff at institutions. In the correctional institution for young men, more women work in treatment, while in the judicial police department the majority of employees are men. In the institution for young women, there are no male employees. In this sense, it is evident that the gender structure of employees reflects gender norms (women as caregivers in typically supportive roles and men in more repressive work roles). The study participants note that employees of institutions hardly allow any questioning of gender and that their behavior promotes gender stereotypes, Participant 1 explained:

“He should be strong, he does not need to show feelings, she should show feelings, but she must not show anger. Because if she gets angry, she ends up in solitary confinement.”

Furthermore, the participants point out that staff at correctional institutions are not aware of gender and gender sensitivity issues and take relatively little interest in them. They believe that this is partly a reflection of a highly gender-stereotyped society, Participant 2 highlighted:

“Because I would relate it to the wider context of society, it, it… there is talking about gender, gender ideology, intimidating people, brainwashing people…we do not talk about gender in school.”

It is also pointed out that correctional institutions and the justice system in general is an environment that is more attractive for employing more conservative people, so the lack of sensitivity to gender issues mentioned is not surprising. Although the system of correctional institutions is generally seen as gender stereotypical, participants note that there are also staff who offer different models and approaches through the design of activities and their attitudes toward young people.

### The characteristics of juveniles in correctional institutions

The third theme concerns the gender-specific characteristics and particularities of young people in correctional institutions. Within this theme, the following codes are defined: specific characteristics of young men, specific characteristics of young women, specific characteristics of the young Roma, diversity of approaches in work related to young people’s gender, and discrimination and prejudice among young people.

Regarding the characteristics of young people in correctional institutions, the focus group participants noted specific characteristics of girls and boys, as well as specific characteristics of young people of the Roma national minority. From the perspective of the focus group participants, a very masculine culture was observed in the groups of young men. Young men are more cautious, even fearful, when it comes to opening and expressing their thoughts and feelings in front of a group of their peers. They choose not to reveal many personal and sensitive things about themselves for fear of being ridiculed by their peers. More intimate and personal topics tend to be treated with humor. The participants report that young men are more direct in their communication with youth workers and use vulgar language more often. A higher level of aggression is also observed among young men. There is a stereotype for young men to be aggressive and violent, which has been shown to be to a higher degree in accordance with the youth worker’s experiences, but not toward them, rather toward their peers. The participants also state that the young men openly expressed their physical competence and perceived masculinity, for example, Participant 1 stated:

“It was more like they went to us to prove something, to do push-ups or something.”

In relation to girls, participants recognize that they open up more easily and are more willing to express feelings. Although the participants recognize gender stereotypes and prejudices in both boys and girls, they believe that girls are more willing to look critically at their thoughts and attitudes and are more willing to change attitudes. According to the participants, they are more open in conversation, show more interest in the topics of the work and ask more questions. They speak very openly about their intimate experiences and enjoy talking about interpersonal relationships, Participant 6 described:

“They asked everything that interested them, without shame, without discomfort. They shared their personal experiences, and very personal experiences. Very, very personal experiences. I would not really expect that from a child.”

It is interesting to note that the focus group participants stated that the staff in the correctional institutions as well as other colleagues prepared them for the fact that “it is more difficult to work with girls,” which they consider a gender stereotype, while their personal experience was different. Participant 1 explained:

“So, the first expectations, the ones we all had, were completely unfounded because it is still wonderful for me to work with girls.”

The participants also point out the specific characteristics of Roma youth and the specific relations, i.e., examples of discrimination against young Roma people by employees of correctional institutions. First of all, it is important to note that young people of Roma origin are relatively overrepresented in correctional institutions. Intersectional vulnerability has been observed in this group of young people, especially among girls. When working with young people of the Roma national minority, it was found that they are often not sufficiently aware of what intimate partner violence is. Young Roma are more likely to have traditional and conservative attitudes toward gender roles and the positions of men and women in the family. The participants also point out that there is discrimination against the Roma national minority by staff, which is expressed in the stricter approach of judicial police officers and in the negative experiences of staff in the context of their social integration after release from the correctional institution, Participant 1:

“We talked about this group of young people and he (staff member) was talking about life perspective of youth…if they will be successful, and in his opinion only young Roma man from this group will not succeed.”

Discrimination is also evident in some positive practices such as the celebration of International Roma Day, when all young people of darker skin shade are congratulated, regardless of whether they belong to the Roma national minority or not.

According to the experiences of the survey participants, young people themselves also have a number of prejudices and discriminatory attitudes toward members of sexual minorities. Young men express more negative attitudes and greater resistance when it comes to issues related to sexual minorities than girls. The participants noted that young men even tended to refrain from participating in certain workshops that dealt with similar topics, Participant 1 described the experience:

“When we differentiated between sex and gender, we got to the point where a group of ten people fell back to the two that were left in the room at the end. Because when we came to that, when we asked if men could wear thongs… And when they asked how they would do it… As a man, I said, "I (youth worker) would wear it like this. And… Six of them left the room.”

Open rejection of gay people was also observed in the institution for men, which can lead to gender-based violence. However, as the participants note, such rigid attitudes change over time and there is more acceptance and understanding.

The focus group participants also commented on the approach to working with girls and boys. They note that there are certain gender-specific preferences in terms of the content, techniques and dynamics of the work. In the correctional institution for men, a slower pace is observed in the development of the relationship between the youth workers and the young people, a slower relaxation and opening of the young men and the realization of a relationship of trust. Girls tend to connect more quickly with the youth workers and are more open to receiving new content. Girls are also more willing to accept creative-expressive techniques than young men. However, participants point out that creative techniques are important when working with young men because they allow them to open up indirectly and talk about topics that make them uncomfortable or are intimate in nature. With young men, it is also important that the art techniques are more adapted to masculine culture, so for example they chose tattoo drawings that they would like to do, for example, Participant 1 stated:

“And with the boys, we tried to look for opportunities that would be interesting for them. So we came up with the idea to give the girls tasks to draw something. With the boys, they did not want to draw, they did not like it. And then we actually came up with the idea of getting a tattoo artist to do their tattoo, so to speak. So, they could draw a tattoo which is really cool for them. And in the end, we use it with girls also because tattoos end up being cool for them.”

Participants also pointed out that it is important to work with young men on the emotions and prevention of violence in partner relationships and bullying in general, while girls need to work more specifically on recognizing violence in partner relationships.

### The characteristics of the programs of external organizations

The fourth theme deals with the characteristics of the programs of external organizations operating in correctional institutions and consists of the following codes: the topics of the programs of external organizations, the relationship of juveniles and staff with external youth workers, misunderstandings between external youth workers and staff of correctional institutions, and recognized needs of juveniles for further treatment work.

Topics of work in external organizations’ programs spanned the spectrum of gender and gender-based violence, sexual and reproductive health, preparation for independent living after leaving care, and artistic expression. Participants pointed out that most of these issues, especially issues related to gender and gender-based violence, are not sufficiently present in the treatment content of the work of institutions. Most staff and young people are not informed about or sensitized about the issues of gender, sexual minorities, gender-based violence, masculinity, and femininity, as could be seen in the previous topics.

An important aspect of the fourth theme is the relationships between employees of correctional institutions and young people and youth workers from external organizations. The youth workers themselves point to their own fears when entering correctional institutions, in terms of how young people will accept them and how they will accept the content they offer. In this sense, women youth workers feared that the young men would see them as potential partners and that they would have no distance from them. In some cases, these fears were unjustified, for example Participant 2 stated:

“I do not think it would have occurred to them… the group I was working with. They were so respectful, as if I was someone’s grandmother.”

But in some cases there were also unpleasant experiences, Participant 3 explained:

“Once a boy said to me about my colleague - so what… she smiles, she likes it - and she smiled because she felt uncomfortable.”

As the participants noted, in some situations, young men expressed their disrespect toward female facilitators, objectifying them and flirting with them, indicating the need to include the topic of male–female relations in the regular content of the work. In the cases where the activities were led by two male youth workers, the participants stated that this was a particularly good experience in the correctional institution for young men, as the young men felt connected to the youth workers and perceived them as older brothers. Furthermore, the participants note that it would be important for the facilitators of the various activities to be people with similar experiences and characteristics as the youth, and it would be especially important to include Roma leaders and more male facilitators. The participants also emphasized that working with young people has made them aware of their own privileges.

The youth workers coming from external organizations are characterized by a greater flexibility in dealing with young people, which they themselves are aware of, especially because they are “outside” the rigid structure of the correctional institutions. They themselves are somewhat of an example and a model for breaking down the usual gender norms. The participants see themselves as those who challenge the existing gender norms in correctional institutions, Participant 4 explained:

“We on the other hand, the aim of our association is to teach them about gender stereotypes, expectations, roles.”

In this sense, certain difficulties can be identified in the relationships and cooperation between external associates and institution staff, especially in situations where youth workers of external organizations have warned about inappropriate actions by correctional institution staff, as Participant 1 stated:

“Now we have reported it and… after that, we have a reaction that is, I would say, inappropriate in a professional relationship…. I doubt we can go any further.”

In the participants’ experience, collaboration between external youth workers and institutions is good until disagreements arise regarding professional behavior and diversity of working approaches. As already stated in the first theme, one of the characteristics of institutions is the rigidity and in terms of accepting criticism, which leads to a reduction or interruption of the activities of external organizers. Unfortunately, this also limits the presence of topics related to gender, gender norms, gender-based violence and the like, which prove to be necessary and relevant topics for young people. From the description of the participants, a certain conflict of approaches and values in working with youth can be inferred, with correctional institutions offering traditional views and gender norms, while external organizations bring flexibility, diversity, and pluralism in terms of content, techniques and approaches to work.

In relation to the characteristics of the programs and working with youth in correctional institutions, the research participants referred in particular to the needs of young people in the context of further treatment work. They are of the opinion that the work programs should be more oriented toward the individual characteristics of the young people rather than being gender-specific, for example Participant 1 stated:

“I would not see it as gender-specific in any way but look at individualized programs for each person separately.”

Both the content of the work and the space in which it is carried out should correspond to the specific individual characteristics of the young people. The participants also suggest introducing more creative elements into the treatment work. In terms of topics, they emphasize the importance of work in the area of partner relationships, especially violence in partner relationships, recognizing and coping with emotions and setting personal boundaries. With regard to gender-specific issues, the participants believe that it is important to work with young men so that they do not feel victimized and do not feel the need to attack others from this position. It is also necessary to work on societal expectations of men and women. They also believe that the work programs of external organizations should be longer-term in order to enable continuity in relationships.

As a particularly important aspect of the work, the research participants mention the training of employees of correctional institutions on the topic of gender and gender norms as well as on the topic of violence. Finally, the participants particularly emphasize the importance of creating an emotionally warm and safe treatment environment in correctional institutions, Participant 4 explained:

“The environment, in the narrow and broader sense, in which we place these issues is what prevents…. If I had a magic wand to change something, I’d change it… Practically the physical environment, but also the program environment… just to expand at least a little bit. That we do not stay on this very narrow straight path.”

## Discussion

The thematic analysis of the focus groups shed light on how youth workers from external associations experience gender norms and the particularities of young people in correctional institutions, as well as how they describe the application of gender-specific approaches in their work.

The results of the analysis showed that the first research question could be answered: “What are the characteristics of correctional institutions in relation to gender norms?,” corresponding to the theme “The rigid structure of correctional institutions” and “Gender stereotypes division of correctional institutions.” The participants describe correctional institutions as institutions with a high degree of structure, security and rules and note that these elements are even more pronounced in male correctional institutions. Correctional institutions are very restrictive and give the impression that the physical environment itself is punitive and not adapted to the needs of young people. In relation to gender norms, the experience in correctional institutions is described as extremely gender stereotypical, which is evident in the physical environment, the range of treatment programs offered, the gender of the staff, and the attitude of the staff toward the youth. The rigidity and high level of structure and gender stereotyping have the effect that traditional gender norms are expressed very stereotypically in everyday life in institutions. In light of the results obtained, it seems important to advocate for the design of physical space in the juvenile justice system that better meets the needs of young people, especially with regard to their age, developmental needs and gender-specific characteristics. A safe correctional environment must include elements of physical and emotional safety (Leone et al., 2017) and attention to developmental needs (Lyman and Campbell, 1996) in order to promote positive behavior change in young people. Even though the juvenile justice system is essentially focused on rehabilitation, research in this area still seems to show that the philosophy of punishment prevails over the philosophy of rehabilitation in daily practice (Sankofa et al., 2018). In addition to the current policy in the field of juvenile justice, it seems important to make additional efforts to monitor the implementation of the policy in the day-to-day functioning of the institutions.

It is also important to emphasize that correctional institutions are slowly opening up to issues related to gender and gender equality through the programs of external organizations that deal with gender and gender norms. The collaboration between external organizations and correctional institutions creates new opportunities to change gender-stereotypical issues, but also stereotypical relationships within correctional institutions. Furthermore, the results show that external youth workers are not sufficiently familiar with the system and the particularities of correctional facilities. In this sense, the results that speak of a low level of understanding and tolerance on the part of external youth workers toward the structure and rules of correctional institutions are particularly noteworthy.

The stereotypical gender standardization is particularly visible but is also supported by staff who have traditional gender expectations of young men and women. Young men are expected not to show emotions and participate in sporting activities, while the environment for girls is less repressive, does not include sporting activities and focuses on traditional female activities such as creative workshops and crocheting. It is particularly important to note the biases that stand out in relation to treatment work, with external youth workers noting that staff consistently describe girls as “more complex and difficult for treatment work”. The findings thus confirm that girls with behavioral problems are more often perceived as more complex and as those who are more difficult to work with (Burman and Batchelor, 2009 Jeđud, 2011; Galardi and Settersten, 2018). In this context, Chesney-Lind and Irwin (2008) speak of gender blaming, in which girls with problems experience greater stigmatization because they violate the gender norms imposed by society. It is important to note that external youth workers challenge attitudes about the complexity and difficulty of working with girls and describe their own experiences of working with girls in very positive terms. The findings therefore suggest that future research should focus on a more comprehensive understanding of the risks, needs and strengths of girls with behavioral problems and that in practice professionals should be empowered to work with girls.

In addition, external youth workers point out that they find that staff in correctional facilities are hardly sensitized to the issues of gender and gender roles and therefore deal with these issues less sensitively. These findings are in line with other research findings with professionals in Croatian correctional institutions who say that they have not had the opportunity to receive training on gender and gender equality and that the need to sensitize correctional officers to gender and other minority issues is particularly felt (Borić and Ćosić, 2022). Similar findings on the need to train staff on gender issues and a gender-sensitive approach are also confirmed by many other studies (Covington and Bloom, 2014; Galardi, 2017; Galardi and Settersten, 2018; Hill, 2019). It is also interesting to note that the gender structure of staff reflects traditional gender norms. Here, external youth workers describe female staff through a typical caring role and male staff through more repressive roles such as judicial police. In this context, Hill (2019) speaks of women in correctional institutions are typically taking on a mix of roles related to care and rehabilitation, leading to the emergence of a feminization of care. When gender roles are very stereotypical in certain environments, young people also have limited opportunities to learn about the diversity of characteristics and behaviors associated with masculinity and femininity.

The second research question was: “What are the specific characteristics of young people in correctional institutions?,” and it is answered by the topic Specific characteristics of young people in correctional facilities.

When it comes to gender roles, participants describe the presence of gender-typical behaviors in girls and boys. Young men are described as more aggressive and vulgar in communication, as those who need more time to build relationships, and as those who are afraid to open up and express thoughts about feelings in front of their peers in the group. On the other hand, girls are described as those who build relationships more easily and are more open to topics related to emotions and interpersonal relationships. These findings are consistent with the expected gender roles that characterize the period of adolescence. Girls are expected to be caring, sensitive and understanding, while in young men vulgarity in communication is tolerated, crying is undesirable and participation in sports activities is encouraged (Galambos, 2004; Jugović and Kamenov, 2008; Ullrich et al., 2022). In the findings, participants also talk about the presence of traditional gender norms and frameworks, which are particularly visible in the differences in terms of the regulation of the environment, staff relationships with juveniles, and the provision of recreational activities and treatment programs. The environment and relationships in correctional institutions are gender stereotypical, which can lead to the transmission of ideas about traditional gender roles and inequality between women and men. Adopting gender-typical behaviors can lead to young men and women learning to have different expectations of men and women, as well as of themselves as individuals and the society in which they live (Ellemers, 2018). In correctional institutions it is necessary to foster a multidimensional and comprehensive treatment environment that empowers girls and boys in a way that focuses on their strengths while promoting at the same time encourages the development of desirable masculine and feminine behaviors. At the same time, promoting masculine and feminine behaviors allows a person to be more adaptable to complex social situations and is consistent with the two-dimensional understanding of the concept of masculinity and femininity, in which the characteristics of both roles can be present in a person simultaneously (Helmreich et al., 1979; Krištof et al., 2013). When conducting activities and group programs, it is important to create a safe environment in which to express one’s own opinions and examine the topic of gender and gender roles. With this in mind, there is a long-term need to invest in programs that promote a positive peer climate in correctional facilities. Positive peer relationships and a positive climate are important elements that can help young people to cope with the hardships in correctional institutions (van der Helm et al., 2009; Eltink et al., 2015; Reid and Listwan, 2018; Sentse et al., 2021).

Furthermore, it has been shown that there is a distinct traditional masculine social climate in the correctional institution for young men which is oriented toward male superiority and aggressive behavior patterns. Prison has not equipped men in such a way that they will be able to readily navigate the structural challenges that they will face on their release (Maguire, 2021.). Rather, the experience of imprisonment has served to intensify the same masculine traits that led to their marginalization and exclusion in the first instance, thereby trapping these men in the revolving-door cycle of imprisonment (Maguire, 2021.). This climate is reflected both in the culture of peers and in relationships, but also in the structure and work organization of the male institution. The results obtained are related to the results of other research in this area, which also show that extreme masculine norms simultaneously promote an aggressive atmosphere, behaviors and relationships in correctional institutions (Flood and Pease, 2009). Bengtsson (2016) emphasizes that these types of institutions for youth are often characterized by a hypermasculine framework of interactions that implies an informal shared understanding of masculinity through superiority, overt sexuality, and a willingness to participate in violence. It is noticeable that the promotion of hyper-masculinity in this setting creates a very limited space for rethinking gender roles and attitudes and promotes gender stereotyping. This is also illustrated by the findings of this research, as external youth workers indicate that both boys and girls rarely question the expectation of a gender role and are unfamiliar with issues related to gender, gender norms and attitudes. In future research, it seems important to focus on a deeper exploration of gender roles and attitudes toward gender roles, especially among young men in correctional institutions, but also among the staff who work with them (educators, judicial police officers and external associates). The results of these studies can be crucial for a deeper understanding of gender norms in prisons and serve as a basis for developing interventions that promote more flexible gender frameworks and tolerance. Staff working with young people and external associates on a daily basis should be particularly aware of their own gender roles and how these are reflected in treatment.

It is noticeable that the participants also emphasize the special characteristics of young Roma. As mentioned in the introduction, the presence of young Roma in correctional institutions is relatively high and, according to the latest research, is 29.2% in the male correctional institution in Croatia (Borić and Ćosić, 2022). While on the one hand this group of young people is strongly represented, this research found that participants talk about disadvantages, i.e., examples of discrimination against young Roma by staff. The findings on the vulnerability and disadvantaged social position of young members of the Roma national minority are in line with other recent research in Croatia that speaks of discriminatory practices and attitudes toward young people of the Roma population (Klasnić et al., 2020; Rašić et al., 2020; Družić Ljubotina et al., 2022). In the context of correctional institutions, it is particularly worrying that discriminatory practices have been observed by staff working with young people on a daily basis. At the same time, some other studies point to the experience of discrimination by other peers with whom they spend time in an educational institution (Ćosić, 2024). This suggests that the treatment environment may pose an additional risk for discrimination and marginalization of certain youth groups, such as young Roma. Treatment interventions in the juvenile justice system should therefore take into account the intersection of different dimensions that can lead to discrimination, such as gender, gender roles and ethnicity. In this regard, it is important that interventions are based on the concept of intersectionality, which is about understanding and adapting work with youth by taking into account the multiple dimensions of interdependent systems of inequality, vulnerability and marginalization in society (Crenshew, 1989). Due to the recognized importance of this concept as a framework for interventions in this system, it is also possible to find examples of interventions that are already based on it (for example, Shade et al., 2011; Haines-Saah et al., 2018; Kelly et al., 2018). Furthermore, the findings suggest that external youth workers speak about the presence of prejudice against sexual minorities, particularly among young men. The findings of this research are in line with previous research in correctional facilities, which indicated that professionals working with young people notice numerous prejudices, especially against homosexual individuals and Roma, and that some young people are not aware that they are victims of discrimination (Borić and Ćosić, 2022). Future research should focus on a deeper understanding of the different bases of discrimination, the attitudes of this group of young people toward sexual minorities, as well as general research on the representation of different sexual minorities in correctional institutions. When developing and implementing treatment programs, it seems extremely important to take into account the extremely negative attitudes and discriminatory behavior of young people toward sexual minorities and members of the Roma national minority.

When it comes to the approach to working with girls and young men, participants point to the different gender preferences in terms of the content, techniques and characteristics of the work. For example, the slower building of relationships and opening up to peers is noticeable in correctional institution for young men, as well as the less frequent use of creative-expressive techniques in the work compared to the girls. External youth workers also describe how they select gender-specific activities and topics depending on whether they are working with girls or young men. The results suggest that gender-specific characteristics should not be neglected or denied when designing interventions in institutions, especially when working directly with young men and women. It is, therefore, necessary for future research to explore similarities and differences in the treatment needs of girls and boys and to use the results obtained to develop scientifically sound interventions. It also seems particularly important to invest in the scientific evaluation of programs that are already being implemented in correctional institutions. Doing so opens up opportunities to move beyond the current one-size-fits-all model of care in a juvenile justice system that fails to address gender-specific risks and needs in treatment programs (Chesney-Lind and Shelden, 2013; Galardi and Settersten, 2018).

The answer to the third research question: “How are gender norms reflected in the work with young people in correctional institutions?,” was obtained through the third and fourth themes called “The characteristics of young people in correctional institutions” and “The characteristics of the programs of external organizations.”

Based on the results, it is clear that external youth workers introduce topics through the implementation of programs that are not sufficiently represented in the regular treatment content of the work of correctional institutions. In this regard, topics related to gender, gender-based violence and sexual and reproductive health stand out. As the external youth workers come from the outside and do not work in correctional institutions, their flexibility and innovation in working with young people are visible. Maguire (2021) explains how teacher who was not constrained by the core prison curriculum and who tailored learning activities to the interests and needs of the room, opened a gateway or an avenue to finding an alternative way of “doing masculinity.” Working with youth on gender and gender equality is an important area of work in this system, as research shows that the gender dimension is very neglected in the juvenile justice context (Abrams et al., 2008). In this area, there is a need to explore more deeply which correctional institutions as treatment environments maintain or change young people’s gender identities, but also to what extent they help young people express their gender identities during their stay in the institution. On the other hand, staff in correctional institutions are poorly informed about gender, sexual minorities, and gender roles, which sometimes leads to distance in cooperation and different approaches to working with youth. It is also important to point out that the young people themselves show resistance to gender issues, especially as they express numerous stereotypes about gender roles. The positive side of the cooperation between external associations and correctional facilities is that external organizations bring in many topics that are not part of the regular treatment programs with young people. The juvenile justice system shows that it recognizes the importance of gender issues, as well as the importance of the work of external associates, because despite the rigid structure and strict rules of the system, they open opportunities to work on gender issues. The results thus suggest that, on the one hand, rigidity, and a gender-stereotypical environment prevail in correctional institutions, while on the other hand, external educators (and programs) bring very liberal attitudes and questioning of gender and gender roles. This discrepancy in the communication and modeling of gender norms between staff and external workers can pose a particular challenge in working with young people and their understanding of issues related to gender and gender equality. By improving collaboration between external associates and employees of the institutions and making all staff more aware of specific ways of working with young women and young men, it is possible to transform institutions from a punitive environment to a multidimensional treatment environment that takes into account the individual needs of these young people.

## Conclusion

The results of this study clearly show the need for a systematic approach and education on gender issues, gender norms and a gender-sensitive approach. First and foremost, the staff of educational institutions (educators and especially judicial police officers) and the young people themselves need to be involved. It would be a good idea to draw on the existing knowledge and resources of external organizations, such as the programs of Status M. Further research is also needed, especially research that examines the attitudes and needs of young people, so that future interventions can be scientifically sound. Through education and further research, it is possible to influence the current status quo in which correctional institutions support gender stereotypes and are not sufficiently sensitive to the specific needs of young people and to different gender expressions.

### Limitations

Although this research has produced some very interesting results and findings in relation to gender, gender norms and gender-sensitive programs in Croatian correctional institutions, it is important to highlight the main limitations. First, this is a qualitative study with a relatively small number of participants and the results cannot be generalized. Furthermore, the participants are external associates of correctional institutions who have only partial insight into the general functioning of these institutions. In future studies, it would be worthwhile to further investigate the perspective of prison staff as well as the perspective of young people.

## Data availability statement

The raw data supporting the conclusions of this article will be made available by the authors, without undue reservation.

## Ethics statement

Ethical approval was not required for the studies involving humans because the research did not involve medical records or human tissue. In the research, participants shared their opinions and experiences regarding gender norms in correctional institutions. In Croatia, the Ethical codex for the research conducted in the field of social sciences and humanities (quantitative, qualitative, interventional and online research) states that ethical approval is needed in case the participants are children or if participants are vulnerable. In this study, participants are not children neither are vulnerable. The participants provided written informed consent to participate in this study. The studies were conducted in accordance with the local legislation and institutional requirements. The participants provided their written informed consent to participate in this study.

## Author contributions

EM: Conceptualization, Data curation, Project administration, Resources, Supervision, Writing – original draft, Writing – review & editing. IB: Conceptualization, Data curation, Formal analysis, Methodology, Writing – original draft, Writing – review & editing. AĆ: Conceptualization, Data curation, Formal analysis, Investigation, Methodology, Writing – original draft, Writing – review & editing. AS: Conceptualization, Data curation, Resources, Supervision, Writing – original draft, Writing – review & editing.

## References

[ref1] Abrams L. S. Anderson-Nathe B. Aguilar J. (2008). Constructing masculinities in juvenile corrections. Men Masculinities 11, 22–41. doi: 10.1177/1097184X06291893

[ref2] Allen D. Hamnett V. (2022). Gypsy, Roma and Traveller children in child welfare Services in England. Br. J. Soc. Work 52, 3904–3922. doi: 10.1093/bjsw/bcab265

[ref3] Bengtsson T. T. (2016). Performing Hypermasculinity: experiences with confined young offenders. Men Masculinities 19, 410–428. doi: 10.1177/1097184X15595083

[ref4] Borić I. Ćosić A. (2022). X-MEN – Masculinities, empathy and non-violence in Croatia. Zagreb: Status M.

[ref5001] Braun V. Clarke V. (2006). Using thematic analysis in psychology. Qualitative Research in Psychology 3, 77–101. doi: 10.1191/1478088706qp063oa

[ref5] Burman M. Batchelor S. A. (2009). Between two stools? Responding to young women who offend. Youth Justice 9, 270–285. doi: 10.1177/1473225409345104

[ref6] Carr N. T. Hudson K. Hanks R. S. Hunt A. N. (2008). Gender effects along the juvenile justice system: evidence of a gendered organization. Fem. Criminol. 3, 25–43. doi: 10.1177/1557085107311390

[ref7] Cénat J. M. McIntee S.-E. Mukunzi J. Noorishad P.-G. (2021). Overrepresentation of black children in the child welfare system: a systematic review to understand and better act. Child Youth Serv. Rev. 120:105714. doi: 10.1016/j.childyouth.2020.105714

[ref8] Chesney-Lind M. Irwin K. (2008). Beyond bad girls: gender, violence, and hype. New York, United States: Routledge.

[ref9] Chesney-Lind M. Shelden R. G. (2013). Girls, delinquency, and juvenile justice. New jersey, United States: John Wiley & Sons.

[ref10] Connell R. W. Messerschmidt J. W. (2005). Hegemonic masculinity: rethinking the concept. Gend. Soc. 19, 829–859. doi: 10.1177/0891243205278639

[ref11] Ćosić A. (2024). Uloga Grupnih Procesa u Subjektivnoj Dobrobiti Adolescenata u Institucijskoj Skrbi. Doctoral dissertation. Zagreb, Croatia: University of Zagreb, Faculty of Education and Rehabilitation Sciences.

[ref12] Covington S. S. Bloom B. E. (2014). “Gender responsive treatment and Services in Correctional Settings” in Inside and out, vol. 29 (Oxfordshire, United Kingdom: Routledge), 9–33.

[ref5002] Crenshew K. (1989). Demarginalizing the intersection of race and sex: A Black feminist critique of antidiscrimination doctrine, feminist theory and antiracist politics. University of Chicago Legal Forum, 140, 139–167.

[ref13] Družić Ljubotina K. Kletečki Radović M. Opačić A. (2022). “Sažetak Studije Slučaja” in Studija o Participaciji Djece iz Ranjivih Skupina u Hrvatskoj. eds. Borić I. Mataga Tintor A. Šalinović M. (Zagreb, Hrvatska: Ured UNICEF-a za Hrvatsku), 148–154.

[ref14] Ellemers N. (2018). Gender Stereotypes. Annu. Rev. Psychol. 69, 275–298. doi: 10.1146/annurev-psych-122216-011719 28961059

[ref15] Eltink E. M. van der Helm P. Wissink I. B. Stams G. J. J. (2015). The relation between living group climate and reactions to social problem situations in detained adolescents: ‘I stabbed him because he looked mean at me’. Int. J. Forensic Ment. Health 14, 101–109. doi: 10.1080/14999013.2015.1033110

[ref16] Flood M. Pease B. (2009). Factors influencing attitudes to violence against women. Trauma Violence Abuse 10, 125–142. doi: 10.1177/1524838009334131 19383630

[ref17] Galambos N. L. (2004). “Gender and gender role development in adolescence” in Handbook of adolescent psychology. New Jersey, United States: John Wiley & Sons. 233–262.

[ref18] Galardi T. R. (2017). Gender matters: The perspectives and experiences of living unit staff in juvenile correctional facilities. Doctoral dissertation. New Jersey, United States: John Wiley & Sons.

[ref19] Galardi T. R. Settersten R. A. Jr. (2018). ‘They're just made up different’: juvenile correctional staff perceptions of incarcerated boys and girls. Child Youth Serv. Rev. 95, 200–208. doi: 10.1016/j.childyouth.2018.10.040

[ref20] Haines-Saah R. J. Hilario C. T. Jenkins E. K. Ng C. K. Johnson J. L. (2018). Understanding adolescent narratives about ‘bullying’ through an intersectional Lens: implications for youth mental health interventions. Youth Soc. 50, 636–658. doi: 10.1177/0044118X15621465

[ref21] Helmreich R. L. Spence J. T. Holahan C. K. (1979). Psychological androgyny and sex role flexibility: a test of two hypotheses. J. Pers. Soc. Psychol. 37, 1631–1644. doi: 10.1037/0022-3514.37.10.1631 512834

[ref22] Hill R. B. (2004). Institutional racism in child welfare. Race Soci. 7, 17–33. doi: 10.1016/j.racsoc.2004.11.004

[ref23] Hill S. (2019). Performing gender and authority: Juvenile corrections Officers' self-perceptions and strategies at work. Doctoral dissertation. Bowling Green State University.

[ref24] Jakupcak M. Tull M. T. Roemer L. (2005). Masculinity, shame, and fear of emotions as predictors of Men's expressions of anger and hostility. Psychol. Men Masculinity 6, 275–284. doi: 10.1037/1524-9220.6.4.275

[ref25] Jeđud I. (2011). Doprinos Perspektive Korisnica Odgojnog doma Bedekovčina Razumijevanju Rizičnosti kod Djevojaka. Doctoral dissertation. Zagreb, Croatia: University of Zagreb, Faculty of Education and Rehabilitation Sciences.

[ref26] Jugović I. Kamenov Ž. (2008). Razvoj Instrumenta za Ispitivanje Rodnih Uloga u Adolescenciji. Suvremena Psihologija 11, 93–106.

[ref27] Juvenile Courts Act. (2019). *Official Gazette*, no. 84/11, 143/12, 148/13, 56/15, 126/19.

[ref28] Kelly M. Barnert E. Bath E. (2018). Think, ask, act: the intersectionality of mental and reproductive health for judicially-involved girls. J. Am. Acad. Child Adolesc. Psychiatry 57, 715–718. doi: 10.1016/j.jaac.2018.07.870, PMID: 30274642 PMC6714969

[ref29] Klasnić K. Kunac S. Rodik P. *Uključivanje Roma u Hrvatsko Društvo: Žene, Mladi i Djeca*. (2020). Available at: https://repozitorij.ffzg.unizg.hr/en/islandora/object/ffzg:5471.

[ref30] Krištof I. O. Maskulinosti F. i Samopoimanja A. (2013). Master thesis, Josip Juraj Strossmayer University of Osijek. Faculty of Humanities and Social Sciences. Osijek, Croatia: Department of Psychology.

[ref31] Leone P. E. Lockwood S. Gagnon J. C. (2017). “Creating and sustaining safe environments in juvenile corrections” in The Wiley handbook of violence and aggression, eds. Peter E. L. Susan L. Joseph C. G.. vol. 1–12.

[ref32] Levant R. F. Richmond K. (2007). A new psychology of men. Washington, D.C, United States: Basic Books.

[ref33] Lyman R. D. Campbell N. R. (1996). Treating children and adolescents in residential and inpatient settings. Thousand Oaks, California: Sage Publications, Inc.

[ref34] Maguire D. (2021). “Male, failed, jailed” in Palgrave studies in prisons and penology (Cham: Springer Nature).

[ref35] Mears D. P. Hwang J. Winterfield L. (2004). Exploring the gender gap: girls in juvenile justice schools and programs. Crime Delinq. 50, 43–61. doi: 10.1177/0011128703261155

[ref36] Ministry of Justice and Public Administration. Report on the condition and work of penitentiaries, prisons, correctional institutions and centers for the year 2022. Zagreb, Croatia: Government of Republic of Croatia. (2024). https://www.sabor.hr/sites/default/files/uploads/sabor/2024-01-05/093102/IZVJESCE_RAD_KAZNIONICA_ZATVORA_ODGOJNIH_ZAVODA_2022.pdf (Accessed February 2, 2024).

[ref37] Moura T. Caruso H. Mascarenhas M. Longo V. Ruíz Garriga Á. Borić I.. (2023). Boys have to step on, or to be stepped on: A comparative study on masculinities, gender, and violence among at-risk youth in Portugal, Spain, and Croatia. Coimbra: CES.

[ref38] Rašić N. Lucić D. G. Galić B. Karajić N. (2020). *Uključivanje Roma u Hrvatsko Društvo: Identitet, Socijalna Distana i Iskustvo Diskriminacije*. Zagreb: Sveučilište u Zagrebu.

[ref39] Rasskazova O. Anholenko V. Kravchenko A. Sulimenko O. Mohylka O. Kuzminykh M. (2021). Readiness of social services specialists for gender-sensitive activities with offence-prone juveniles. Journal of Educational and Social Research. 11, 48. doi: 10.36941/jesr-2021-0050

[ref40] Reid S. E. Listwan S. J. (2018). Managing the threat of violence: coping strategies among juvenile inmates. J. Interpers. Violence 33, 1306–1326. doi: 10.1177/0886260515615143, PMID: 26598293

[ref41] Ricijaš N. Mandić S. Lamešić L. (2022). Dynamics of juvenile delinquency in the Republic of Croatia from 2000 to 2020. Criminol. Soci Integ. 30, 70–104. doi: 10.31299/ksi.30.1.4

[ref42] Sankofa J. Cox A. Fader J. J. Inderbitzin M. Abrams L. S. Nurse A. M. (2018). Juvenile corrections in the era of reform: a Meta-synthesis of qualitative studies. Int. J. Offender Ther. Comp. Criminol. 62, 1763–1786. doi: 10.1177/0306624X17727075, PMID: 28831820

[ref43] Sentse M. Kreager D. A. Bosma A. Q. Nieuwbeerta P. Palmen H. (2021). Social Organization in Prison: a social network analysis of interpersonal relationships among Dutch prisoners. Justice Q. 38, 1047–1069. doi: 10.1080/07418825.2019.1700298

[ref44] Shade K. Kools S. Weiss S. J. Pinderhughes H. (2011). A conceptual model of incarcerated adolescent fatherhood: adolescent identity development and the concept of intersectionality. J. Child Adolesc. Psychiatr. Nurs. 24, 98–104. doi: 10.1111/j.1744-6171.2011.00274.x, PMID: 21501286

[ref45] Ullrich R. Becker M. Scharf J. (2022). The development of gender role attitudes during adolescence: effects of sex, socioeconomic background, and cognitive abilities. J. Youth Adolesc. 51, 2114–2129. doi: 10.1007/s10964-022-01651-z, PMID: 35838855 PMC9508033

[ref46] Van der Helm P. Klapwijk M. Stams G. Van der Laan P. (2009). ‘What works’ for juvenile prisoners: the role of group climate in a youth prison. J. Children's Serv. 4, 36–48. doi: 10.1108/17466660200900011

